# Automated and Efficient Generation of General Molecular Aggregate Structures

**DOI:** 10.1002/anie.202214477

**Published:** 2022-12-16

**Authors:** Christoph Plett, Stefan Grimme

**Affiliations:** ^1^ Mulliken Center for Theoretical Chemistry Clausius-Institut für Physikalische und Theoretische Chemie Universität Bonn Beringstraße 4 53115 Bonn Germany

**Keywords:** Global Optimization, Non-Covalent Interaction, Quantum Chemistry, Supramolecular Chemistry

## Abstract

Modeling intermolecular interactions of complex non‐covalent structures is important in many areas of chemistry. To facilitate the generation of reasonable dimer, oligomer, and general aggregate geometries, we introduce an automated computational interaction site screening (aISS) workflow. This easy‐to‐use tool combines a genetic algorithm employing the intermolecular force‐field xTB‐IFF for initial search steps with the general force‐field GFN‐FF and the semi‐empirical GFN2‐xTB method for geometry optimizations. Compared with the alternative *CREST* program, aISS yields similar results but with computer time savings of 1–3 orders of magnitude. This allows for the treatment of systems with thousands of atoms composed of elements up to radon, *e.g*., metal‐organic complexes, or even polyhedra and zeolite cut‐outs which were not accessible before. Moreover, aISS can identify reactive sites and provides options like site‐directed (user‐guided) screening.

## Graphical abstract

A completely automated, efficient, and easy‐to‐use tool for generating accurate dimers up to large aggregate structures is presented. This algorithm, termed aISS, can be applied to molecules of any size and across the periodic table, which allows combined with its high efficiency the accurate modeling of systems not possible before.

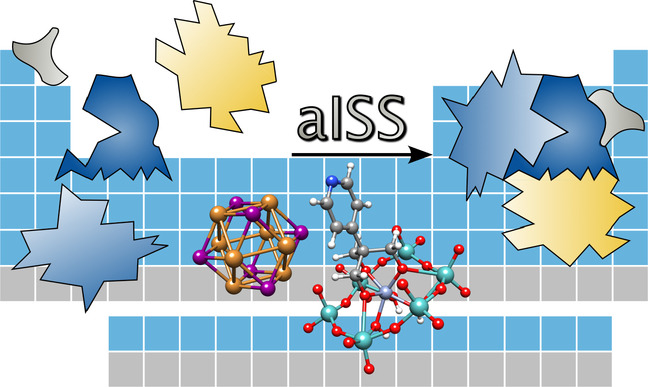

## Introduction

The question of how molecules interact non‐covalently and arrange geometrically in an optimal way is of fundamental relevance for many chemical systems and plays an important role in various physical, biological, and chemical processes.^[1]^ It is crucial for the association and function of supermolecular dimers and oligomers,^[2]^ the self‐assembly of molecules,^[3]^ or protein‐protein and protein‐ligand complexes[^4^, ^5^] to name only a few examples. Nowadays, computational studies often complement experimental work to elucidate structure and interaction (free) energies in great detail^[6]^ and finally even predict complex reaction mechanisms.^[7]^ Usually, well‐established wave‐function theory (WFT) or Kohn–Sham (KS) density functional theory (DFT) are used as computational methods for simulating basic molecular properties, but they quickly reach their limits for the structure generation of large oligomer and aggregate systems containing a few hundred atoms due to high computational costs. This encouraged the development of various semi‐empirical and force field methods that extend the treatable system size drastically.[^8^, ^9^] Recent examples are the semi‐empirical GFN*n*‐xTB^[10]^ molecular orbital, tight‐binding methods, as well as the generally applicable force field GFN‐FF,^[11]^ which proved to provide reasonable Geometries, Frequencies, and Non‐covalent interactions (GFN).[^12^, ^13^, ^14^] If only non‐covalent interactions (NCIs)[^15^, ^16^] are of interest, specialized approaches can be used that describe exclusively intermolecular interactions^[17]^ and otherwise treat the molecules as rigid fragments. One of these methods is the intermolecular force field xTB‐IFF,^[18]^ which uses specifically pre‐computed electronic properties from xTB for the two interacting molecular fragments to improve the theoretical description without introducing too much computational overhead. Even though all these methods can be used to optimize and analyze intermolecular structures, they cannot generate them efficiently for which special algorithms are required. Typically, existing software solutions for docking molecules focus on protein‐ligand binding,^[19]^ which is either based on simple scoring functions for binding affinities or on a very simplified calculation of the interaction energies between the fragments.[^20^, ^21^] Commonly used algorithms can either do rigid docking, (e.g., *ZDOCK*,^[22]^ and *RDOCK*),^[23]^ flexible‐rigid docking, (e.g., *Flex X*,[^24^, ^25^] *AutoDock*,^[26]^ and *Autodock Vina*),^[27]^ or fully flexible docking like *Gold*,^[28]^ *Glide*,^[29]^ and *LeDOCK*.^[30]^ These highly specialized algorithms have a limited range of applications (typically restricted to biomolecules) and cannot be applied to general chemistry. Until now, a universal and easy‐to‐use method that is capable of finding interaction sites and generating reasonable dimer, oligomer, and aggregate structures for molecules with sizes up to several thousand atoms and arbitrary elemental composition is missing. To close this gap, we here introduce a robust and efficient algorithm for this purpose, named automated interaction site screening (aISS). It enables the investigation of intermolecular geometries for a wide variety of systems like transition‐metal catalysts, zeolites, or MOFs that were inaccessible before due to their size or chemical composition. The freely available aISS algorithm is designed to be easily applicable also by computational non‐experts and provides useful additional features like site‐directed (user‐guided) screening. After a short description of the algorithm, aISS is tested for chemically interesting example systems and the interaction energies of the resulting structures are re‐evaluated with high‐level DFT methods.

### aISS Method

**Figure 1:**
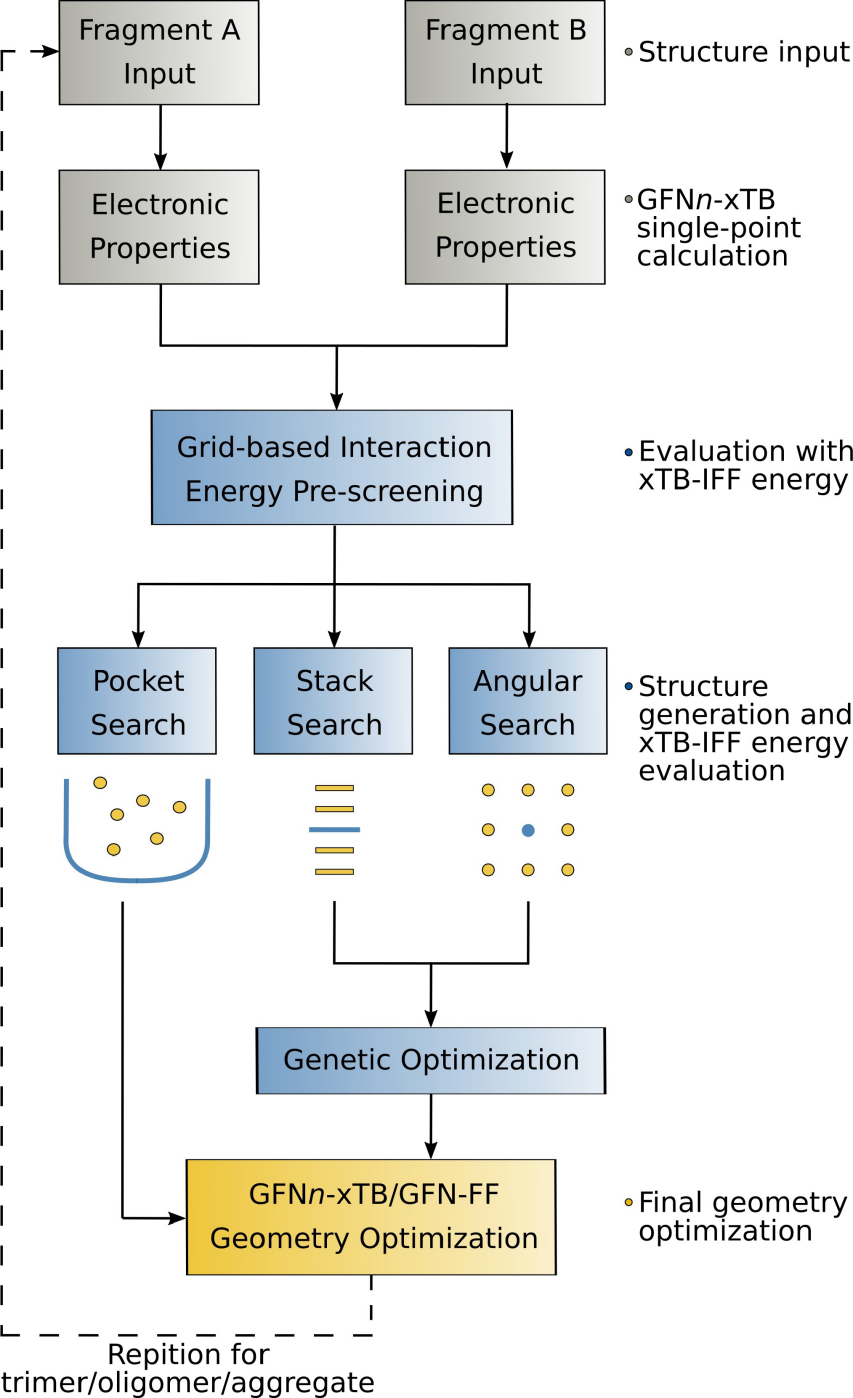
Schematic depiction of the aISS algorithm.

The principal idea of aISS is to find the energetically lowest structure (global energy minimum) of the largest interaction between two given fragments, which often has a dominant impact in the real system. A few energetically higher structures (per default 15) are also obtained by the algorithm and optionally, a more complete ensemble of thermally populated structures can be generated. As the possible bonding motifs and geometrical structures can be rather diverse and complex, multiple steps applying different approximations are performed during an aISS run (Figure 1).

The automated algorithm is invoked with one simple command‐line keyword. Only two sets of input coordinates for the molecular fragments to be combined are required but information about charges, unpaired electrons, and different geometrical constraints can be provided as well. The procedure starts with GFN*n*‐xTB computations of the electronic properties of fragments A and B which are needed for the xTB‐IFF interaction energy calculations. This step is done only once as the information can be used for any generated intermolecular geometry of the fragments A and B. Next, a grid‐based xTB‐IFF energy pre‐screening is conducted around fragment A with a neutral and artificially +/−0.1 charged rare‐gas atom (Kr), which allows a fast exploration of possible interaction sites. The following three steps are independently used for generating different intermolecular geometries from the rigid fragments. Thereby, fragment B is moved around fragment A and the structures are evaluated in terms of the xTB‐IFF energy. Fragment A will always be the first molecular geometry file given in the program call. It is recommended to provide the larger fragment first, as the screening process will generally be more efficient if the smaller fragment is moved around the larger one. Two of the structure‐generating steps aim for typical bonding motifs: a search for pockets in fragment A and a screening for π‐π‐stacking interactions along different directions in three dimensions (3D). The third structure‐generating step is a search for general orientations of fragment B on an angular grid around fragment A including the best positions of the Kr atom pre‐screening. The resulting structures of the pocket search are fully optimized including all intramolecular degrees of freedom with GFN*n*‐xTB or GFN‐FF, while the energetically lowest structures (per default 100) of the stack and angular search are combined and refined by a two‐step genetic algorithm.^[31]^ This ensures the inclusion of positions and orientations that are not yet covered by the grid‐based searches. During this genetic optimization, first, a random crossover of each pair of positions of fragment B around fragment A is done according to:

(1) $$\sum_{i=1}^{N}\sum_{j=1}^{N}\left[\left(\begin{array}{c} \begin{array}{c} \begin{array}{c} x_{ij}\\ y_{ij} \end{array}\\ \begin{array}{c} z_{ij}\\ \alpha_{ij} \end{array}\\ \beta_{ij} \end{array}\\ \gamma_{ij} \end{array}\right)=\left(\begin{array}{c} \begin{array}{c} \begin{array}{c} x_{i}\cdot f_{1}\\ y_{i}\cdot f_{2} \end{array}\\ \begin{array}{c} z_{i}\cdot f_{3}\\ \alpha_{i}\cdot f_{4} \end{array}\\ \beta_{i}\cdot f_{5} \end{array}\\ \gamma_{i}\cdot f_{6} \end{array}\right)+\left(\begin{array}{c} \begin{array}{c} \begin{array}{c} x_{j}\cdot \left(1-f_{1}\right)\\ y_{j}\cdot \left(1-f_{2}\right) \end{array}\\ \begin{array}{c} z_{j}\cdot \left(1-f_{3}\right)\\ \alpha_{j}\cdot \left(1-f_{4}\right) \end{array}\\ \beta_{j}\cdot \left(1-f_{5}\right) \end{array}\\ \gamma_{j}\cdot \left(1-f_{6}\right) \end{array}\right)\right],$$

where *x*, *y*, and *z* are Cartesian coordinates that describe the center of mass (CMA) of fragment B with respect to the CMA of fragment A, *α*, *β*, and *γ* are the three Euler rotational angles of fragment B and *f*_1_–*f*_6_ are random numbers between zero and one. The second step of the genetic optimization is a random mutation of 50 % of the structures in position and angle. The xTB‐IFF energy of each newly generated structure is used for ranking and the genetic step is repeated per default ten times (for further details, see the Supporting Information). Finally, either a few energetically lowest geometries (per default 15) or all structures with significantly attractive xTB‐IFF interaction energy are optimized, depending on if just a single structure or an ensemble is requested. For these geometry optimizations, either the default GFN2‐xTB (aISS//GFN2‐xTB), or alternatively GFN1‐xTB (aISS//GFN1‐xTB) or GFN‐FF (aISS//GFN‐FF) can be chosen. Optionally, they can be conducted including a standard continuum solvation model.^[32]^ These optimizations ensure intramolecular relaxation of the rigidly added fragments and refinement of the entire complex. If not only a dimer, but a trimer or oligomer is desired, the dimer geometry can be used as an input for a subsequent run to either add a third molecule or to combine two dimers. This can be repeated iteratively with different fragments until a desired size and composition of the aggregate is reached. Because the GFN2‐xTB electronic property calculations are conducted for fragments of increasing size, polarization as well as other many‐body effects are accounted for already at this intermolecular force‐field level thereby increasing accuracy and physical reliability. To ensure the free availability and easy application of the aISS algorithm, we implemented it in the open‐source *xtb*[^10^, ^33^] program and provided a detailed documentation.^[34]^

## Results and Discussion

### General Applicability and Evaluation

**Figure 2:**
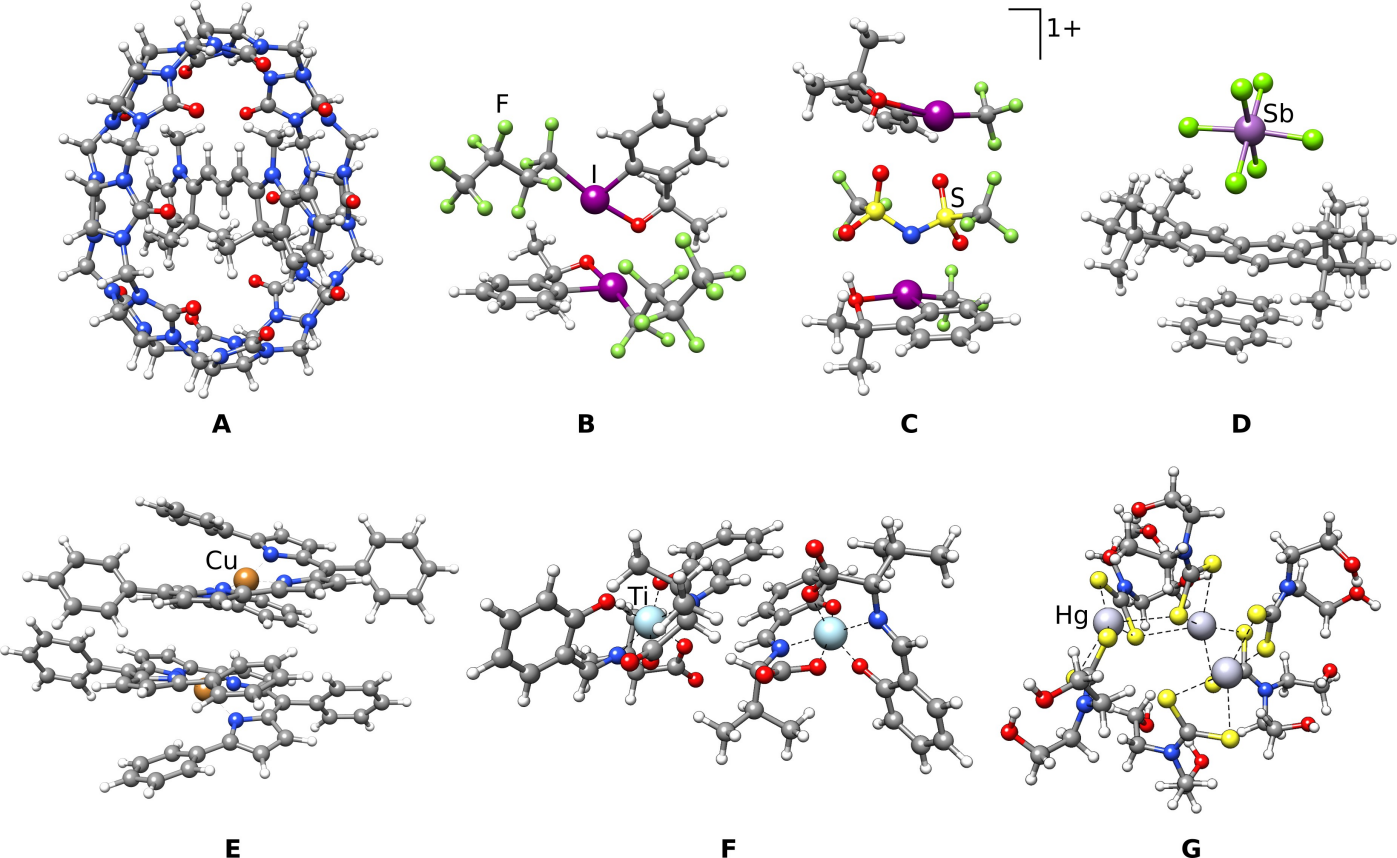
The lowest energy structures for an example set of complexes resulting from the aISS algorithm.

To demonstrate the general applicability and efficiency of the aISS algorithm and to validate the resulting structures, we investigated a variety of currently researched dimers and trimers (Figure 2).[^35^, ^36^, ^37^, ^38^, ^39^, ^40^]

These small to medium‐sized systems contain up to 300 atoms including heavy main‐group elements and transition metals, which are usually challenging or not even included for common force fields that do not employ electronic information. Thus, the default GFN2‐xTB^[41]^ was chosen for the final geometry optimizations in the aISS algorithm (aISS//GFN2‐xTB). For evaluating the performance, a comparison with the more elaborated but well‐tested and established NCI‐iMTD workflow of the Conformer‐Rotamer Ensemble Sampling Tool (*CREST*)[^42^, ^43^] is given, which is based on metadynamics^[44]^ and molecular dynamics simulations to find different conformers. GFN2‐xTB was also employed for this NCI‐iMTD workflow of *CREST*. For a quantitative comparison, the interaction energies (*E*_int_) for the energetically lowest GFN2‐xTB structures are compared. Because both algorithms search for the global minimum‐energy structure, the most attractive (negative) interaction energy corresponds to the best result. For GFN2‐xTB (and alternatively for GFN‐FF or DFT) this value was calculated as the difference in total energy of the fully relaxed monomers (*E*(*A*) and *E*(*B*)) and the dimer (*E*(*AB*)), i.e., *E*_int_=*E*(*AB*)−*E*(*A*)−*E*(*B*). Additionally, r^2^SCAN‐3c^[45]^ interaction energies were calculated after geometry re‐optimization of the GFN2‐xTB structures to validate the results with a very accurate approach for NCIs and conformational energies. Therefore, the Commandline Energetic Sorting of Conformer Rotamer Ensembles (*CENSO*)^[46]^ program was employed to filter and to re‐optimize the ensembles resulting from either the NCI‐iMTD workflow of *CREST* or the ensemble run‐type of the aISS algorithm, respectively. For the single‐structure aISS run‐type, only the best GFN2‐xTB structure was re‐optimized. The resulting interaction energies are shown in Table 1 as absolute values and as percentages of *E*_int_ relative to the NCI‐iMTD results for comparison together with the timings on a usual desktop computer.

**Table 1:** GFN2‐xTB and r^2^SCAN‐3c (DFT) interaction energies in kcal mol^−1^ of the molecules shown in Figure 2. The structures were generated with the aISS algorithm in ensemble (aISS^E^) and single‐structure (aISS^S^) run‐type. For comparison, interaction energies for structures generated with the NCI‐iMTD workflow of the *CREST* program are shown together with computational timings on 14 cores of an Intel® Xeon® CPU E5‐2660 v4 @ 2.00 GHz.

	**A**	**B**	**C**	**D**	**E**	**F**	**G**
$E_{\mathrm{int}}^{\mathrm{GFN}2-\mathrm{xTB}}$ (*CREST*)	−62.0	−29.3	−153.0	−84.0	−26.1	−39.1	−95.0
$E_{\mathrm{int}}^{\mathrm{GFN}2-\mathrm{xTB}}$ (aISS^S^)	−62.0	−26.6	−153.7	−83.4	−22.9	−29.2	−101.5
	(100 %)	(91 %)	(101 %)	(99 %)	(88 %)	(75 %)	(107 %)
$E_{\mathrm{int}}^{\mathrm{GFN}2-\mathrm{xTB}}$ (aISS^E^)	−62.0	−28.3	−155.4	−83.5	−25.5	−36.0	−109.5
	(100 %)	(97 %)	(102 %)	(99 %)	(98 %)	(92 %)	(115 %)
$E_{\mathrm{int}}^{r^{2}\mathrm{SCAN}-3c}$ (*CREST*)	−61.1	−12.1	−114.2	−119.2	−23.3	−23.9	−50.9
$E_{\mathrm{int}}^{r^{2}\mathrm{SCAN}-3c}$ (aISS^S^)	−60.6	−11.7	−115.3	−119.2	−21.5	−23.7	−57.2
	(99 %)	(97 %)	(101 %)	(100 %)	(92 %)	(99 %)	(112 %)
$E_{\mathrm{int}}^{r^{2}\mathrm{SCAN}-3c}$ (aISS^E^)	−61.1	−12.1	−119.8	−121.9	−22.4	−24.1	−54.4
	(100 %)	(100 %)	(105 %)	(102 %)	(96 %)	(101 %)	(106 %)
Comp. time (*CREST*)	64 h 23 min	5 h 3 min	2 h 9 min	12 h 30 min	45 h 2 min	17 h 15 min	32 h 4 min
Comp. time (aISS^S^)	15 min	3 min	8 min	4 min	7 min	5 min	11 min
Comp. time (aISS^E^)	13 h 57 min	2 h 35 min	4 h 32 min	5 h 19 min	9 h 36 min	9 h 22 min	27 h 52 min

For the cationic trimethine cyanine within the cucurbit[8]uril (Figure 2A),^[35]^ the interaction energies of the aISS structures are almost identical to those from the NCI‐iMTD algorithm. This indicates a similar structure quality and holds also for the electronically difficult cationic, open‐shell octamethylated‐naphthalene complex between naphthalene and anionic antimony hexafluoride (Figure 2D)^[37]^ and the paramagnetic copper complex (Figure 2E).^[38]^ Noteworthy is the tremendous difference in computational time. While the *CREST* runs take days to weeks (up to 64 h 23 min), the aISS runs complete within a few minutes. The examples of the perfluoroorganyl iodine dimer (Figure 2B),^[36]^ and the titanium complex in Figure 2F,^[39]^ show a somewhat less attractive GFN2‐xTB interaction energy for the aISS structures, but upon employing r^2^SCAN‐3c for *E*_int_ they become almost identical again. This shows that the aISS single‐structure run‐type is able to find reasonable structures, even for cases where the GFN2‐xTB potential energy surface deviates from the usually more realistic DFT energy surface. The example of two perfluoroorganyl iodine cations and one bistriflimide anion (Figure 2C),^[36]^ reveals another advantage of the aISS over MD‐based approaches. During the *CREST* run, the electronically complex monomers had to be constrained to avoid covalent bond breaking in the molecules during the biased (high‐energy) metadynamics simulations, while for the aISS algorithm no further constraints had to be set. The *CREST* run led therefore to a less relaxed structure compared to the aISS result as seen in a slightly more attractive *E*_int_ for the latter. The imposed restriction is also notable in the *CREST* simulation time that is only in this case smaller compared to the aISS ensemble run‐type. If more complex systems with multiple molecules like the mercury‐complex trimer (Figure 2G)^[40]^ are modeled, the conformational space is often too large to find the global minimum‐energy geometry within reasonable MD simulation times.^[47]^ In such cases, the more systematic aISS algorithm yields better results in only a tiny fraction of computational time. In comparison, the r^2^SCAN‐3c geometry optimizations took between 1 h 18 min (Figure 2D) and 8 h 31 min (Figure 2E). Hence, if DFT‐optimized geometries are required, this can become the computational bottleneck, especially for the *CREST* and aISS ensemble approaches, where many structures are optimized.

**Figure 3:**
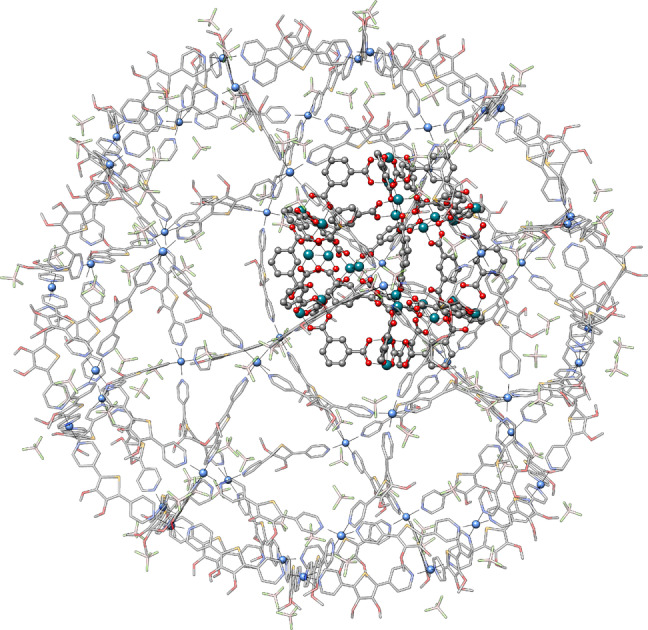
The best structure of the rhodium‐organic cuboctahedra inside the Pd_48_L_96_(BF_4_)_96_ Goldberg polyhedron found with the aISS//GFN‐FF algorithm. Hydrogen atoms are omitted for clarity. Pd is depicted in light blue, Se in orange, B in pinkish, and Rh in light sea green.

The high efficiency of aISS, especially in combination with GFN‐FF geometry optimizations (aISS//GFN‐FF) instead of GFN2‐xTB, together with its universal applicability enables the treatment of systems that were not possible before. As an example, a rhodium‐organic cuboctahedra^[48]^ was added into the largest self‐assembled Goldberg polyhedron Pd_48_L_96_(BF_4_)_96_.^[49]^ The resulting structure (Figure 3) contains 4296 atoms in total, which makes other computational approaches like *CREST* unfeasible due to their much higher cost. Moreover, the system is composed of borate anions, selene, palladium, and rhodium, which excludes the application of other current docking software focusing on biomolecules. The total computation time on 14 cores of a usual desktop computer amounts to just 29 h, where the searching of interaction sites and the generation of the structures took 1 h while the final GFN‐FF geometry optimizations took about 24 h.

To further highlight the general applicability, we treated the single‐chain proteins Barnase and Barstar^[50]^ with the aISS//GFN‐FF algorithm employing the ALPB water solvation model.^[32]^ Different from common docking software for biomolecules, no manual atomic charge assignment or other preparation had to be done as the required properties are automatically generated by the GFN2‐xTB single‐point calculations of the fragments. The energetically lowest, GFN‐FF‐optimized Barnase‐Barstar dimer resulting from the aISS algorithm (Figure S4 in the Supporting Information) shows a reasonable interaction geometry, similar to the one observed in the X‐ray structure.^[51]^ Its GFN2‐xTB interaction energy in water, calculated as the difference between the electronic energies including solvation of the dimer and the isolated monomers in the dimer geometry, amounts to a realistic value of −143.1 kcal mol^−1^.

### Reactive Sites

**Figure 4:**
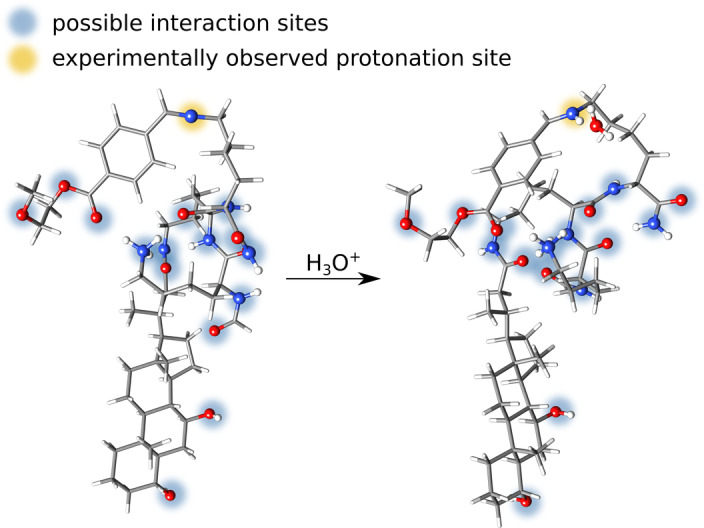
Addition of oxonium to the micelle. Possible interaction sites are marked in blue. The experimentally observed protomer found correctly by aISS is marked in yellow.

With the aISS//GFN2‐xTB workflow, also real chemical reactions are accessible if the energy barriers from the initially found NCI complexes are small enough to be overcome during the final GFN2‐xTB geometry optimizations. This simplifies the identification of reactive sites. For demonstration, the protonation of a micelle cut‐out was investigated (Figure 4).^[52]^ These kinds of biomolecular structures gained a lot of attention due to their possible use as an anti‐tumor drug targeting system. From the experiment, it is known that the benzoic imine moiety (Figure 4) is the pH‐sensitive linker and gets cleaved reversibly in acidic solutions.^[52]^ To explore this behavior, aISS was used with the implicit ALPB water solvation model^[32]^ to add an oxonium ion to the micelle cut‐out to simulate acidic conditions. Even though the substrate exhibits many different basic interaction sites like alcohol, ether, amino, and aldehyde functionalities, the result shows that not only the correct protonation position was found, but also the experimentally observed proton transfer occurred during the subsequent GFN2‐xTB geometry optimization. Thus, realistic modeling of small‐barrier reactions can easily be done with the aISS.

### Directed Interaction Site Screening

**Figure 5:**
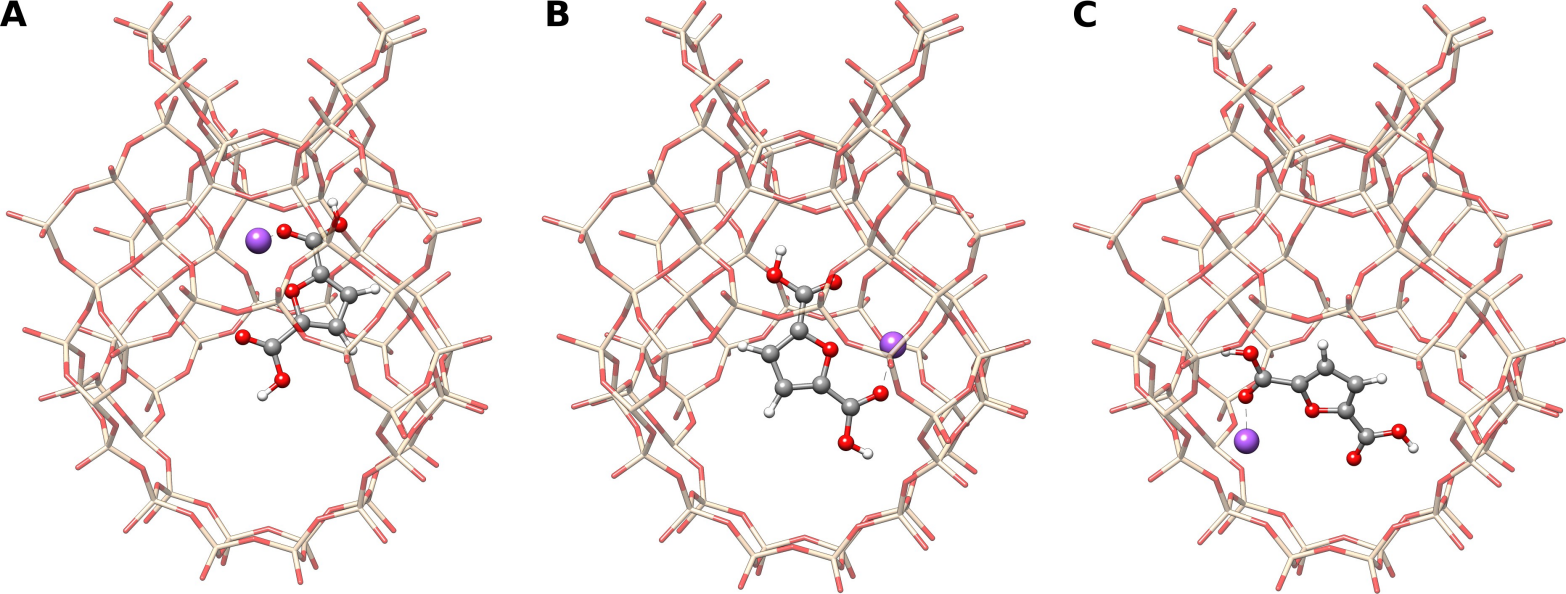
Favored interaction site (A) and two directed interaction sites (B, C) of a sodium cation and a tetrahydrofuran‐2,5‐dicarboxylic acid at a faujasite‐based zeolite according to the aISS//GFN2‐xTB algorithm. Hydrogen atoms are omitted for clarity. Sodium cations are depicted in purple, silicon in beige.

Another unique feature of the aISS algorithm is the directed addition of molecules to certain functional groups and sites defined by the user. Hence, it becomes possible to study interactions at possibly active sites of a molecule that must not be energetically most favored or to restrict the search space according to chemical knowledge or interest. This can be done in two ways: by either adding a distance‐dependent repulsive potential to the atoms outside a user‐defined region or by adding an attractive potential to the desired interaction site. Both bias potentials are considered only for the xTB‐IFF energy screening to prevent unphysical structures after the subsequent geometry optimization. The repulsive potential is useful for strongly interacting molecules with large interaction sites, e.g., large biomolecules like proteins and DNA. Here, an attractive potential would possibly result in a too short distance between the two fragments as the strong attraction is further enhanced. The attractive potential is preferred for weakly interacting or sterically crowded molecules like stereoselective catalysts. Applying the repulsive potential to systems with such sterically crowded interaction sites might lead to a complete repulsion of the added fragments. As an example, different interaction sites of a faujasite zeolite (Figure 5) are investigated for catalytic activity. These kinds of catalysts offer different confinements for exchangeable sodium cations that can be used for Diels–Alder reactions. Thereby, the sodium placement on multiple accessible sites does play an important role in the course of different Diels–Alder reactions.^[53]^ These sites can be modeled easily by performing a directed placement of the sodium cations first and then adding the substrate. Fixing the cut‐out geometry of the zeolite in the aISS//GFN2‐xTB run ensures that the crystal structure geometry is kept. Some of the resulting complexes are shown in Figure 5. The different positions of the sodium cation directly influence the orientation of the substrate, which will result in spatially and electronically different surroundings. This causes different GFN2‐xTB interaction energies: −206.7 kcal mol^−1^ for the non‐directed docking (Figure 5A), while the other positions yield energies of −201.1 kcal mol^−1^ (Figure 5B) and −192.9 kcal mol^−1^ (Figure 5C).

**Figure 6:**
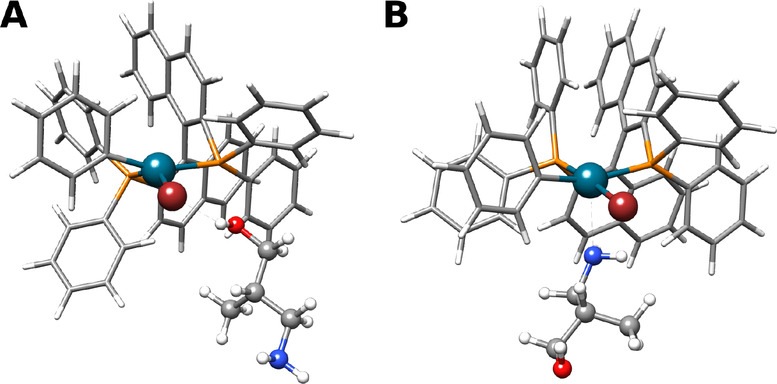
Favored interaction site (A) and to the amine directed interaction site (B) according to the aISS//GFN2‐xTB algorithm. Pd is depicted in dark turquoise, Br in dark red, and P in orange.

Another example is from the field of transition‐metal catalysis: the Buchwald Hartwig amination of bromobenzene and (S)‐3‐amino‐2‐methylpropan‐1‐ol with a Pd(BINAP) catalyst.^[54]^ After the oxidative addition of bromobenzene to the catalyst, the amine approaches the palladium center for the following reductive elimination. Modeling this intermediate without the use of the directed interaction site screening leads to the formation of a halogen bond between bromine and the alcohol moiety (Figure 6A). To focus on the amine group, the attractive bias potential of the aISS algorithm can be used. This leads to a low‐energy structure with the amine directly bound to the palladium of the catalyst (Figure 6B), as required for the further course of the reaction. Hence, the directed interaction site screening can not only be used to investigate different adsorption sites, but also to generate realistic geometries for the clarification of reaction mechanisms.

## Conclusion

The generation of general dimer, oligomer, and aggregate geometries is a challenging problem for computational chemists. With aISS, a general and robust automated interaction site screening workflow is presented that efficiently generates physically reasonable intermolecular geometries of molecules containing thousands of atoms with elements up to radon. It is easily applicable, requires only fragment input coordinates, and can iteratively be employed to investigate even complex reaction mixtures. For medium‐sized test systems, the generated structures are comparable in quality (and sometimes even better) than those from elaborated MD‐based searching methods. The huge computational time savings enable the investigation of systems with several thousand atoms that could not be treated before. Moreover, reactive sites can be identified and low‐barrier reactions like protonations can be modeled with aISS//GFN2‐xTB. One of the unique features is the directed interaction site screening of molecules at certain regions or functional groups, allowing the treatment of user‐defined reactions or binding sites. A computer program implementing aISS can be download free of charge^[33]^ and detailed instructions on how to use the program can be found online.^[34]^

## Conflict of interest

There are no conflicts to declare.

1

## Supporting information

As a service to our authors and readers, this journal provides supporting information supplied by the authors. Such materials are peer reviewed and may be re‐organized for online delivery, but are not copy‐edited or typeset. Technical support issues arising from supporting information (other than missing files) should be addressed to the authors.

Supporting Information

Supporting Information

## Data Availability

The data that support the findings of this study are available in the supplementary material of this article.
