# Supplementary material for: Automated and Efficient Generation of General Molecular Aggregate Structures
Source: Angew Chem Int Ed Engl. 2022 Dec 16;62(4):e202214477. doi: 10.1002/anie.202214477 (PMC10107477; doi:10.1002/anie.202214477)
Supplement: Supplementary file 1 — Supporting Information [file ANIE-62-0-s002.pdf]

Angewandte Chemie
Eine Zeitschrift der Gesellschaft Deutscher Chemiker

Supporting Information

Automated and Efficient Generation of General Molecular Aggregate Structures

*C. Plett, S. Grimme**

Supporting Information

Section S1 Supplementary Text

Section S2 Availability

Section S3 Computational Details

Section S4 Detailed aISS Algorithm

Section S5 Number of Genetic Optimization Steps

Section S6 Protein Example

Section S1: Supplementary Text

In the supporting information (SI), the details of the aISS algorithm are explained and further computational details are provided as well as all necessary data to reproduce the results from this work. For a complete description of the technical implementation (which can not be given here due to its huge complexity), the reader is referred to the free xtb source code.[1]

Section S2: Availability

The *xtb*[1] program package is available free of charge. A detailed introduction on how to use the software is provided online.[2] Collected atomic Cartesian coordinates (XYZ format) are available in addition to the Supporting Information.

Section S3: Computational Details

The aISS algorithm was applied as implemented in the *xTB* program.[1] Unless stated otherwise, the default settings were employed throughout. The *CREST* program[3] was applied as version 2.12 that used *xTB* version 6.4.1.[4] Geometry optimizations and single-point

calculations with r^2SCAN-3c were carried out with *TURBOMOLE* rev. 7.5.1,[5–7] which was also employed by the *CENSO* 1.2.0 program.[8] Default settings of both programs were used. Visualization of the molecules was performed with the *UCSF Chimera* (version 1.15) program[9] and pictures of workflows were generated with *Inkscape* 1.2.[10] Each structure used was previously GFN2-xTB optimized, except for the rhodium-organic cuboctahedra, the $Pd_48L_96(BF_4)_96$ Goldberg polyhedron, and the faujasite-zeolite. Every calculation in this work was performed on 14 cores of an Intel(R) Xeon(R) CPU E5-2660 v4 @ 2.00GHz.

Section S4: Detailed aISS Algorithm

To invoke the aISS algorithm implemented in *xTB*, a simple command line argument in the form of *xtb dock <coordinates A> <coordinates B> [options]* is sufficient. Accepted coordinate formats are xyz format, MDL MOL format, Structure Data File format, Protein Data Bank format, DFTB+ gen format, *Gaussian* external format and *TURBOMOLE* format. The options block is optional and can be used to switch on an implicit solvent model, to provide information about charges and unpaired electrons, to determine the method for final geometry optimizations, or to request an ensemble. An optional file for additional settings can be provided and specified with the command line using "–input <file name>". Here, geometrical constraints, the directed interaction site screening, additional potentials, and parameters can be set. A detailed overview of options can be found in the documentation.[2] During the aISS run, first, both fragments are transformed to have their center of mass (CMA) placed at the origin. The next step is the calculation of GFN2-xTB atomic partial charges, charge centers of LMOs, frontier orbitals, and orbital energies of fragments A and B that are required by the xTB-IFF. The following pre-screening with a krypton atom is done on a grid whose size depends on the extent of fragments A and B in 3D. For this pre-screening, only xTB-IFF Pauli repulsion, dispersion, and electrostatic energy are taken into account and also a $+$ and $-$ 0.1 charge on the Kr atom is evaluated. For the subsequent

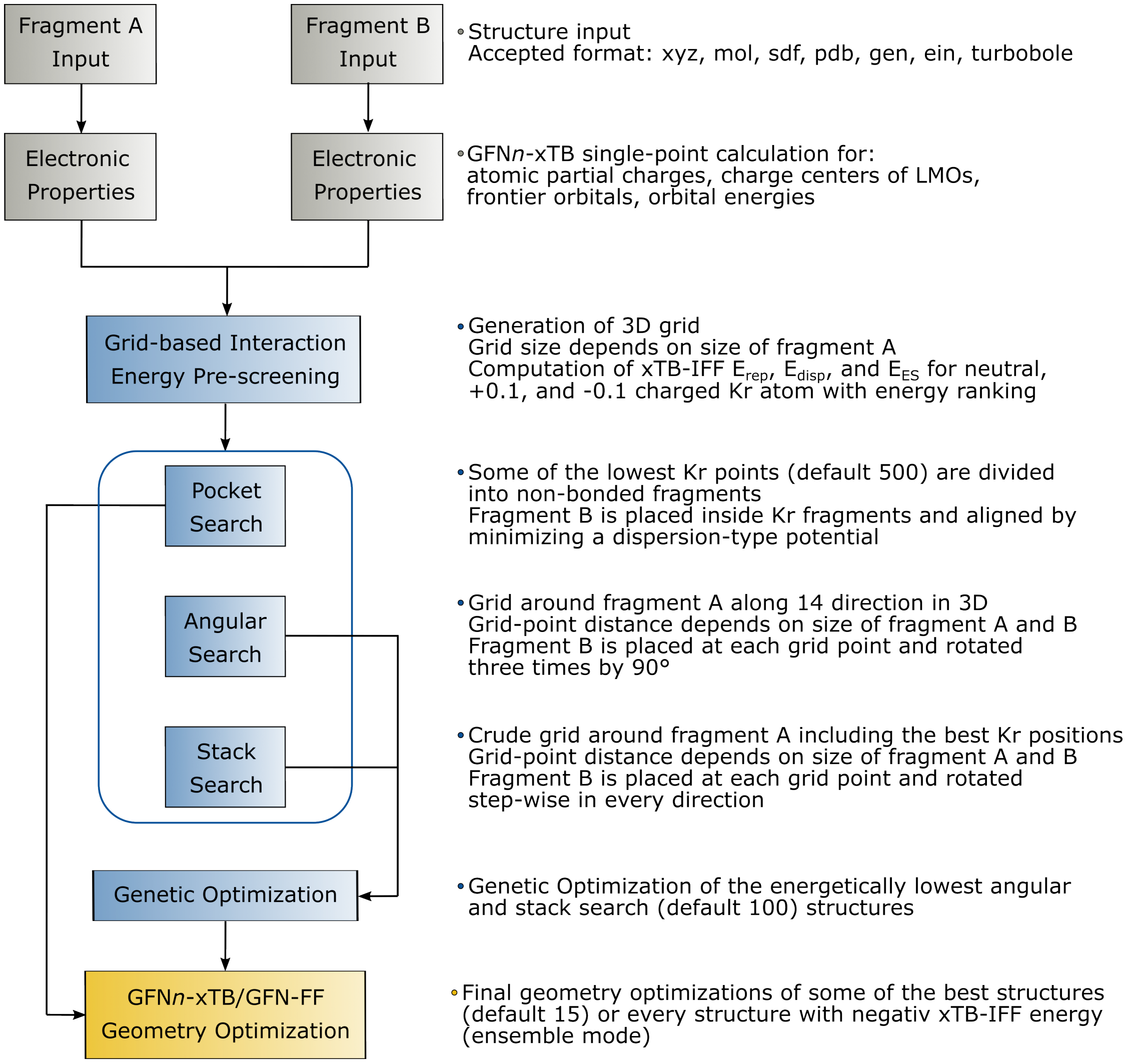

Figure 1: Detailed depiction of the aISS workflow.

search for pockets, per default, the 500 energetically lowest neutral Kr points on the grid are recursively split into non-bonded fragments (if possible) and fragment B is aligned to these fragments by fitting a dispersion-type potential during a genetic optimization. The different orientations of fragment B are combined with fragment A and optimized with GFN*n*-xTB or GFN-FF. Next, the angular search generates different points in 14 directions around fragment A whose distance depends on the spatial extent of fragments A and B. Fragment B is then placed at each position and rotated three times around 90° to account for different π–π-stacking interactions. The xTB-IFF interaction energy is computed for each geometry. Then, a general grid with 64 points, again dependent on the spatial extent

of fragments A and B, is generated for the angular search including the best pre-screened positions with the $+/-$ 0.1 charged Kr atom. Fragment B is placed on each of the positions and step-wise rotated along the three Euler rotation angles. Again, the xTB-IFF energy of every geometry is computed. Per default, the 100 structures with the lowest xTB-IFF energies of the stack and angular search are used for the genetic optimization, described in the article. After this, the energetically lowest structures (per default 15) are used for the final GFN*n*-xTB or GFN-FF geometry optimizations. Alternatively, every structure with negative xTB-IFF energy is optimized, if an ensemble is requested. The method for geometry optimizations can be chosen between GFN-FF, GFN1-xTB, and GFN2-xTB (default) and can also be applied with the implicit ALPB or GBSA solvation model.[11] Two files will result, one with only the best structure in XYZ format and one with every unique, optimized geometry.

Section S5: Number of Genetic Optimization Steps

In the following, the impact of the number of genetic optimization steps is shown with a few structures (Figure2) that were generated with the aISS algorithm.

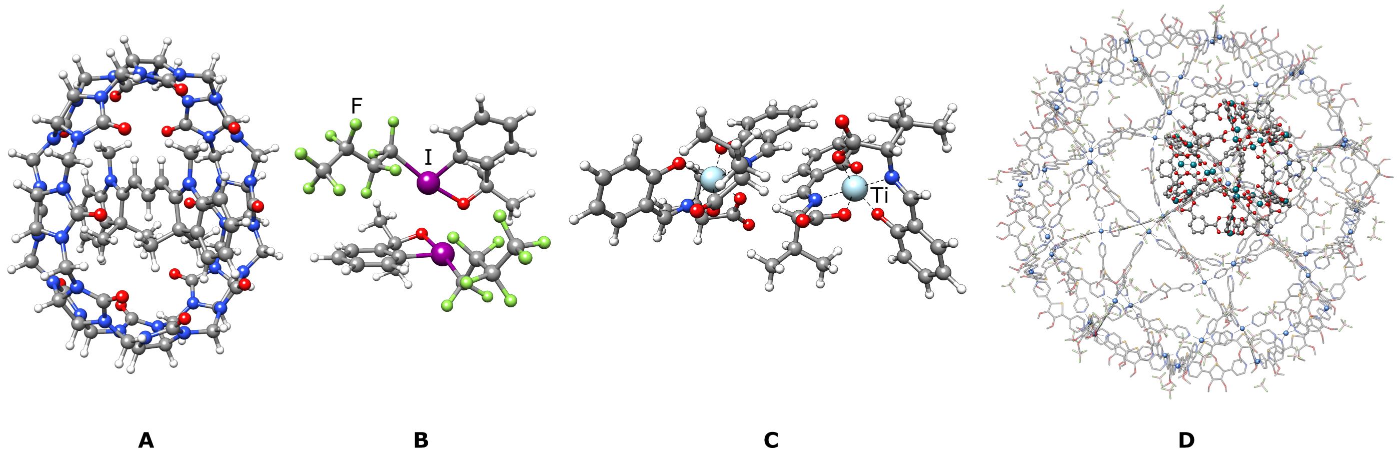

Figure 2: Structures used to show the impact of different numbers of genetic optimization cycles. In structure **D**, hydrogen atoms are omitted for clarity and Pd is depicted in light blue, Se in orange, B in pinkish, and Rh in light sea green.

For each of the structures, 20 independent aISS runs were performed with 50 genetic optimization steps per run. For each of these optimization steps, the change in xTB-IFF energy was computed and referenced to the final xTB-IFF interaction energy obtained after the 50 steps. The resulting percentages of the energy changes were averaged over the 20 runs per structure and are shown in relation to the respective genetic optimization step in Figure 3.

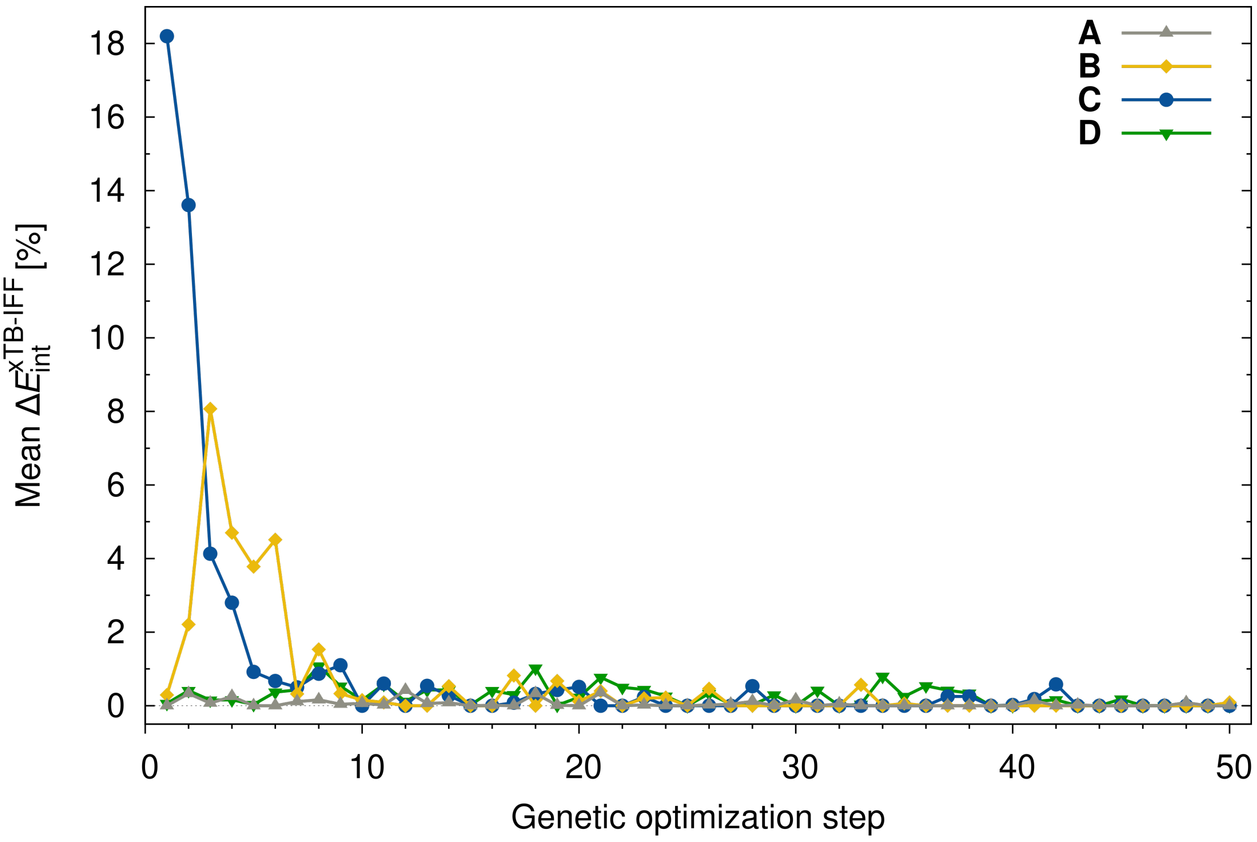

Figure 3: Mean change of the xTB-IFF interaction energies after each genetic optimization step averaged over 20 aISS runs. Shown are the percentages relative to the xTB-IFF interaction energy obtained after 50 steps.

Generally, the most significant changes of the xTB-IFF interaction energy and thus the largest changes in geometry occur for the first few genetic optimization steps but diminish quickly. It can be seen that the ideal number of repetitions is not only influenced by the size, but also by the complexity of the non-covalent interaction motifs. For example, the largest molecule (Figure 2, **D**) has overall the lowest percentual changes as the geometry

after the structure screening is already almost converged in terms of xTB-IFF interaction energy. For systems with large structural changes upon genetic optimization, the most significant differences can be observed for up to ten genetic optimization steps. Thus, the static default value for this number was chosen to be ten. It can be easily adjusted by the user for more complex systems with the *–maxgen* keyword.

Section S6: Protein Example

Figure 4 depicts the energetically lowest structure resulting from the aISS//GFN-FF algorithm with the implicit ALPB water model[11] employed to the single-chained proteins Barnase and Barstar.[12]

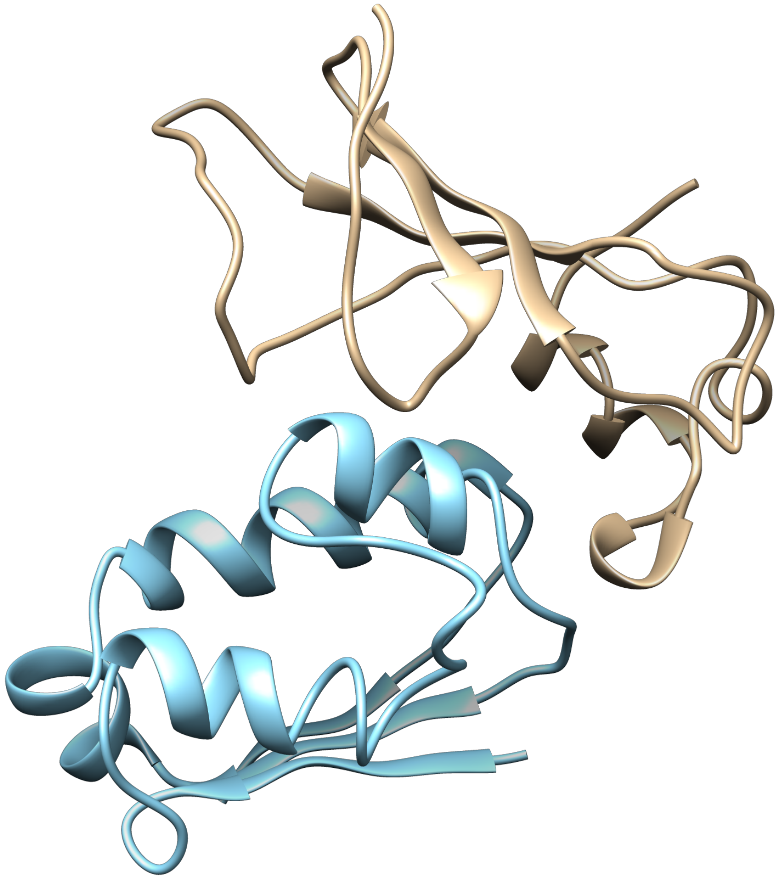

Figure 4: The best structure of the Barnase-Barstar protein complex found with the aISS//GFN-FF algorithm.

Acknowledgement

This work was financially supported by the Merck KGaA. The authors thank Dr. M. Bursch, Dr. S. Ehlert, T. Gasevic, Dr. A. Hansen, and Dr. S. Spicher for helpful discussions.

References

(1) *Semiempirical Extended Tight-Binding Program Package*, **2022**, https://github.com/grimme-lab/xtb.

(2) *Documentation for xtb and related software*, **2022**, https://xtb-docs.readthedocs.io/.

(3) *Conformer-Rotamer Ensemble Sampling Tool based on the xtb Semiempirical Extended Tight-Binding Program Package V2.12*, **2022**, https://github.com/crest-lab/crest/releases/tag/v2.12.

(4) *Semiempirical Extended Tight-Binding Program Package V6.4.1*, **2021**, https://github.com/grimme-lab/xtb/releases/tag/v6.4.1.

(5) F. Furche, R. Ahlrichs, C. Hättig, W. Klopper, M. Sierka, F. Weigend, *Wiley Interdiscip. Rev. Comput. Mol. Sci.* **2014**, *4*, 91–100.

(6) R. Ahlrichs, M. Bär, M. Häser, H. Horn, C. Kölmel, *Chem. Phys. Lett.* **1989**, *162*, 165–169.

(7) *TURBOMOLE V7.5.1* ***2020****, a development of University of Karlsruhe and Forschungszentrum Karlsruhe GmbH, 1989-2007, TURBOMOLE GmbH, since 2007; available from http://www.turbomole.com.*

(8) *CENSO - Commandline ENergetic SOrting of Conformer Rotamer Ensembles V1.2.0*, **2022**, https://github.com/grimme-lab/CENSO/releases/tag/v.1.2.0.

(9) E. F. Pettersen, T. D. Goddard, C. C. Huang, G. S. Couch, D. M. Greenblatt, E. C. Meng, T. E. Ferrin, *J Comput. Chem.* **2004**, *25*, 1605–1612.

(10) *Inkscape Project V1.2*, **2022**, https://inkscape.org.

(11) S. Ehlert, M. Stahn, S. Spicher, S. Grimme, *J. Chem. Theory Comput.* **2021**, *17*, 4250–4261.

(12) R. W. Hartley, *Trends in Biochemical Sciences* **1989**, *14*, 450–454.
